# NICE: an evolving institution

**DOI:** 10.1308/003588413X13511609957650

**Published:** 2013-03

**Authors:** Michael Rawlins

When the National Institute for Health and Clinical Excellence (NICE), or the National Institute for Clinical Excellence as it was then known, was first established in 1999 its responsibilities were twofold. It was charged with providing guidance to the National Health Service (NHS) on the use of (mainly new) health technologies (so-called technology appraisals) on the basis of their clinical and cost effectiveness. It was also expected to develop clinical guidelines, again taking account of effectiveness and cost effectiveness. Since that time, NICE’s responsibilities have expanded substantially and these now encompass four separate activities:

1. developing and publishing *guidance* for NHS health professionals and those with responsibilities for the wider public health;
2. preparing and disseminating *performance standards and metrics* for those providing and commissioning care for NHS patients;
3. offering a range of *information services* for the NHS and for those providing public health and social care services; and
4. using a variety of approaches to encourage the *implementation* of NICE guidance.

## Guidance

As of November 2012, NICE has published 852 individual pieces of guidance (Table 1). This takes several forms:

**Table 1:** NICE guidance: 1999 – November 2012

Guidance product	Published	In development
Technology appraisals	267	142
Clinical guidelines	153	63
Interventional procedures	434	30
Public health	41	29
Medical technologies	11	10
Diagnostics	7	7

*Technology appraisals*: In this programme, NICE continues to assess the clinical and cost effectiveness of health technologies, as it has since 1999. These are predominantly new pharmaceutical products but have included devices, procedures and diagnostic agents.

*Clinical guidelines*: These provide NHS healthcare professionals with advice on the diagnosis and management of individual conditions. They are systematically developed recommendations to assist professional and patient decisions about appropriate care in specific clinical circumstances.

*Interventional procedures*: This programme ****provides guidance on whether (mainly new) interventional procedures are effective and safe enough for use in the NHS. It does not examine an intervention’s cost effectiveness although a procedure for which a positive recommendation has been made may subsequently undergo a technology appraisal or be incorporated in specific clinical guidelines. More information about this programme was published in the October 2012 issue of the *Annals*.^1^

*Public health*: Since 2005, NICE has provided guidance, based on effectiveness and cost effectiveness, to the wider public health community. This guidance covers measures concerning disease prevention, health improvement and health protection, both in and outside of the NHS (eg local and central government, schools and workplaces).

*Cost saving medical technology reviews*: Begun in 2009, ****this programme evaluates cost saving medical technologies and helps facilitate their access to and use in the NHS. It has been designed specifically to align with the NHS’s resource constrained environment and aims to identify promising new products that do not increase cost pressures. Recent medical technologies guidance relevant to surgeons has already been described in this journal.^2^

*Assessment of diagnostic agents*: This relatively new programme assesses the clinical and cost effectiveness of (again, mainly new) diagnostic agents including both in vitro and imaging modalities.

## Performance standards and metrics

NICE now develops three forms of performance standards and metrics based largely on its clinical guidelines:

1. *Quality and Outcomes Framework (QOF)*: NICE undertakes the development of an annual menu of potential indicators for inclusion in the clinical component of the QOF. NICE also recommends whether existing indicators should continue or be retired.
2. *Quality standards*: These are a concise set of between 6 and 8 statements, with accompanying metrics that are designed to drive and measure priority quality improvements in a particular area of care. They have also been described, in greater detail, in this journal previously.^3^
3. *Clinical Commissioning Group Outcomes Indicator Set*: NICE is developing a framework for measuring the health outcomes and quality of care achieved by clinical commissioning groups (CCGs). This will allow the NHS Commissioning Board to identify the contributions of individual CCGs in achieving the priorities for health improvement identified in the health secretary’s ‘mandate’ for the NHS.

## Information services

NICE prepares (or commissions) a range of services so that health and social care professionals have ready access to reliable information.

*NHS Evidence*: This is an online search engine that identifies material that is relevant to a particular clinical problem. As part of the service, NICE also provides access to information content, purchased on behalf of the NHS, including a range of bibliographic databases and professionals journals.

*NICE Pathways*: Merging the totality of NICE guidance can be a challenge for all healthcare professionals. NICE Pathways collects together and displays in algorithmic form the totality of NICE guidance about a particular topic or condition, making it easier for clinicians to look across the care pathway.

*NICE guidance ‘apps’*: All NICE’s guidance products are now available as applications or ‘apps’ for smartphones and tablets, providing ready access to guidance in a form that suits many healthcare professionals.

*British National Formulary (BNF) and the British National Formulary for Children (BNFC)*: These are published jointly by the Royal Pharmaceutical Society and the British Medical Association under contract to the NHS through NICE. In its recent contract negotiations, NICE has ensured that the developers provide the BNF and BNFC in a way that allows NHS staff free access through apps on smartphones and tablets.

*Medicines and prescribing support*: It would ****impractical for NICE to try to undertake technology appraisals for all new indications, formulations and preparations of marketed pharmaceutical products. NICE therefore produces *Evidence Summaries: New Medicines* and *Evidence Summaries: Unlicensed/Off-label Medicines.* The former **provides information about new products while the latter offers information about the use of medicines outside their licensed indications.

## Implementation

NICE has a programme to support the adoption of NICE guidance. Its implementation team works alongside guidance developers, the communications directorate and the field-based staff to ensure that NICE guidance and support is known about and acted on within the NHS and local government. In addition to providing costing and educational tools to support putting guidance into practice, the implementation programme also monitors the uptake of NICE guidance.

There is a legal obligation on primary care and NHS hospital trusts to provide products for which NICE has published a positive technology appraisal if a clinician considers it in the best interests of an individual patient. No such obligation is placed on individual clinicians in respect of any NICE guidance. Indeed, it would be impossible to prepare guidance covering every individual encounter between a patient and his or her clinician. Nevertheless, NICE does expect all its guidance to be taken fully into account by clinicians when looking after NHS patients.

## The future

NICE was established originally in 1999 as a special health authority. It will be re-established on 1 April 2013 under the provisions of the Health and Social Care Act 2012 as a non-departmental public body called the National Institute for Health and Care Excellence (still referred to as NICE). This change in legal status, however, will not have any significant bearings on its existing roles and responsibilities, and healthcare professionals, generally, are unlikely to notice the new statutory arrangements.

NICE will, nevertheless, become responsible for providing two new guidance products. It will begin the publication of guidelines for children’s and adults’ social care together with associated quality standards.^3^ Many of us hope that this will help resolve at least some of the tensions that have existed, between the two services, for too long. NICE will also become responsible for developing guidance on the use of ‘very high cost low volume’ products such as enzyme replacement treatments for the very rare liposomal storage diseases.

The roles and responsibilities of NICE will, undoubtedly, continue to evolve over the years. Its reputation for using the best available evidence – in the practice of safe, effective and cost effective medicine and surgery – has many other applications and NHS patients are entitled to the benefits they bring.
